# Gene mutational analysis by NGS and its clinical significance in patients with myelodysplastic syndrome and acute myeloid leukemia

**DOI:** 10.1186/s40164-019-0158-5

**Published:** 2020-01-06

**Authors:** Jifeng Yu, Yingmei Li, Tao Li, Yafei Li, Haizhou Xing, Hui Sun, Ling Sun, Dingming Wan, Yanfang Liu, Xinsheng Xie, Zhongxing Jiang

**Affiliations:** Department of Hematology, The First Affiliated Hospital of Zhengzhou University, 1 East Jianshe Road, Zhengzhou, 450052 China

**Keywords:** Next generation sequencing, Myelodysplastic syndrome, Acute myeloid leukemia, Gene mutation, Induction chemotherapy, Clinical outcome

## Abstract

**Background:**

In this study, we retrospectively summarized the differences of molecular gene mutations between MDS and AML patients, as well as the young and older age groups of MDS and AML patients. We also analyzed the response of newly diagnosed AML patients to standard DA or IA induction chemotherapy and the relationship between the chemotherapy outcome and the frequency of different gene mutation abnormalities.

**Methods:**

NGS assay covering 43 genes was studied in 93 de novo MDS and 325 non-M3 AML patients. Bone marrow samples from all patients underwent gene mutational analysis by NGS.

**Results:**

At least one non-synonymous gene mutation was detected in 279 AML patients (85.8%) and 85 MDS patients (91.4%). Contrary to 59 years and younger AML patients, there was a significantly higher incidence of gene mutation in 60 years and older AML patients (2.37 vs 1.94, p = 0.034). Gene mutation incidence in 60 years and older MDS patients increased, but no statistical significance was present (1.95 vs 1.64, p = 0.216). AML patients had a significantly higher gene mutation incidence compared with MDS-MLD patients (2.02 vs 1.63, p = 0.046). Gene mutation incidence was higher in patients with MDS-EB1/EB2 compared with patients with MDS-MLD but there was no statistical significance present (2.14 vs 1.63, p = 0.081). AML patients had significantly higher incidences of CEBPA, FLT3-ITD, DNMT3A, NPM1 and IDH1/2 gene mutations (p = 0.0043, 0.000, 0.030962, 0.002752, and 0.000628, respectively) and a lower incidence of TET2 and U2AF1 gene mutations (p = 0.000004 and 0.000, respectively) compared with MDS patients. Among the individual genes in different age groups, there were significantly higher incidences of RUNX1, IDH2, TP53 and SF3B1 gene mutations (p = 0.0478, 0.0028, 0.0024 and 0.005, respectively) as well as a trend of higher ASXL gene mutation (p = 0.057) in 60 years and older AML patients compared to 59 years and younger patients. There was no statistically significant difference in MDS patients with the different age groups and among the individual genes. Between AML patients and MDS patients among the different gene functional groups, AML patients had a significantly higher incidence of transcriptional deregulation (27.4% vs 15.1%, p = 0.014963), activated signalling (36.3% vs 10.8%, p = 0.000002) related gene mutations as well as a significantly lower incidence of RNA spliceosome (6.15% vs 60.1%, p = 0.000) related gene mutations. Furthermore, among the patients who received either IA or DA regimen for induction chemotherapy, patients with IA regimen had a significantly better CR rate than those with DA regimen (76.6% vs 57.1%, p = 0.0228).

**Conclusions:**

Different gene mutations had been found in majority of MDS and AML patients. MDS and AML patients had different gene mutation patterns. AML patients with fewer or no gene mutations had a better chance of achieving CR when treated with IA and DA regimen induction chemotherapy.

## Introduction

Myelodysplastic syndromes (MDS) are a group of progressive clonal disorders which comprise a heterogeneous group of hematopoietic stem cell diseases categorized by dysplasia in one or more hematopoietic cell lineages, as well as cytopenia and functional abnormalities in bone marrow lineages [1–4]. Many studies have shown MDS leads to an increased risk of transformation to acute myelogenous leukemia (AML) [5]. Transformation from MDS to AML often involves clonal evolution or expansion of existing subclones that can be assessed by changes in variant allele frequencies of the somatic mutations that define them. There are a number of predictors for transformation that have been identified, which include mutations of genes in growth signaling pathways (NRAS, KRAS, PTPN11, FLT3-ITD), mutations in genes more commonly observed in AML (NPM1, WT1, IDH2), and certain cytogenetic abnormalities (monosomy 7, complex karyotype, loss of 17p). Gene expression profiles identify a progenitor gene signature subtype associated with a high risk of AML transformation. Assessing for these genetic abnormalities may better identify MDS patients at greatest risk of transformation [6]. Moreover, an increasing number of somatic mutations have been identified in MDS [7–12]. However, most of the current literature used NGS panels with 30–50 genes [13, 14], though the optimal target gene panel and the impact of panel size are not well-defined for these studies.

Acute myeloid leukemia (AML) is a malignant clonal disease originating from myeloid progenitor or multi-potential progenitor cells. It is a molecular heterogeneous disease with a variety of molecular biological abnormalities. Various clonal disorders of AML result from the failure of differentiation and uncontrolled proliferation of hematopoietic progenitor cells. At the same time, many different cytogenetic disorders and gene mutations can accumulate.

In the last decade, significant progress has been made expanding the mutational landscape of AML [15–17], mainly due to advances in sequencing techniques. The recent advances of next-generation sequence (NGS) have made it more practical for clinical research to explore the cytogenetic analysis in many different diseases, including AML. With the advances of chemotherapy, most AML patients can achieve complete remission (CR) after receiving the standard regimen of first-line of chemotherapy. The addition of chemotherapy, hematopoietic stem cell transplantation, immunotherapy, and molecular targeted therapy to traditional forms of treatment allows many patients to achieve longer remission-free survival times. Currently, the standard treatment of daunorubicin and cytarabine (DA) or idarubicin and cytarabine (IA) induction chemotherapy is still recognized as the preferred first-line treatment for AML. The CR rate of first-line treatment of this standard treatment regimen is 60–70% in young adults and 40–50% in older adults [17]. It has been found that the cytogenetic disorder is an important factor in addition to other factors, such as old age, poor performance status and concomitant comorbidity, which are related to outcomes in older AML patients [18–20]. Recently, a combination of cytogenetic analysis and mutation testing has been integrated into the classification and risk assessment of AML patients [21–23]. In 2017, European Leukemia Net revised the prognostic model for AML by adding RUNX1 and ASXL1 mutations to the previously identified molecular risk categories, which included mutations in NPM1, CEBPA, FLT3–ITD and TP53. With this classification and risk assessment model, AML patients can be stratified into three prognostic groups: good, intermediate and poor risk. Many studies show that molecular alterations occur in AML patients with both young and older age groups [23, 24]. Moreover, some studies have demonstrated different chromosome abnormalities and gene mutation patterns among older AML patients [21, 22, 24].

In this study, we retrospectively summarized the differences of molecular gene mutations between MDS and AML patients, as well as the young and older age groups of MDS and AML patients. We also analyzed the response of newly diagnosed AML patients to standard DA or IA induction chemotherapy and the relationship between the chemotherapy outcome and the frequency of different gene mutation abnormalities.

## Methods

### Patient cohort

Ninety-three patients were newly diagnosed with de novo MDS at the First Affiliated Hospital of Zhengzhou University (Zhengzhou, China) between January, 2016 and March 2019. Diagnosis and classification of MDS was based on the multidisciplinary approach which integrates morphology, immunophenotyping, cytogenetics and molecular biology according to WHO classification [1, 25].

325 adult patients were newly diagnosed with non-M3 AML at the First Affiliated Hospital of Zhengzhou University (Zhengzhou, China) between January 2016 and March 2019. The diagnosis and classification of AML was based on the multidisciplinary approach integrating morphology, immunophenotyping, cytogenetics and molecular biology in the presence of ≥ 20% of blasts in bone marrow aspirates according to the FAB criteria and WHO classification [25–27]. Bone marrow samples from all 325 patients underwent gene mutational analysis by NGS. Informed consent was obtained from all patients and the protocol was approved by the Ethics Committee of the First Affiliated Hospital of Zhengzhou University.

### Cytogenetics and fusion genes analysis

Bone marrow samples were studied using G-banding analysis and karyotyped according to the International System for Human Cytogenetic Nomenclature. Fusion genes mutational status was determined by real-time PCR (RT-PCR). Multiplex RTPCR Fusion Gene Kits provided by Rightongene were used.

### Next generation sequencing

Gene mutation was done with standard Second-generation sequencing technology on a Illumina MiSeq System (Illumina, San Diego, CA) high-throughput sequencing platform.

### Library preparation

The extracted DNA was interrupted by fragmentation into the same size and addition of adapters at 5′ and 3′. This linker is mainly used to improve PCR efficiency and provides barcode/index when sequencing.

### Patient cohort generating clusters

Single-sequence sequences are amplified into clusters by bridge PCR. Sequencing: Each fragment is read by a machine based on the principle of SBS (Synthesis by Side Synthesis).

Data comparison and analysis: Read pairs were aligned to Refseq hg19 (downloaded from the UCSC Genome Browser, URLs) by Burrows–Wheeler Aligner version 0.7.13-r1126. Samtools version 1.3 was used to generate chromosomal coordinate-sorted BAM files. We used targeted next-generation sequencing with a Rightongene AML/MDS/MPN Sequencing Panel (Rightongene).

The NGS libraries were paired-end sequenced (2 × 150 bp) on an Illumina MiSeq System (Illumina, San Diego, CA). The mean depth of each sample was 2500×, with an average 98% of the target sequence covered sufficiently deep for variant calling. detection sensitivity was ~ 5% (a mutation with a variability of 5% or more can be reported). SAMtoolsMpileup was applied for SNV/indel calling and filter workflow.

Based on Sequencing by Synthesis (SBS) technology, the Illumina MiSeq System (Illumina, San Diego, CA) high-throughput sequencing platform sequenced libraries to produce large amounts of high quality data. Analyses were conducted of the relevant mutations of 22 genes, including FLT3-ITD, NPM1, KIT, CEBPA, DNMT3A, IDH1, IDH2, TET2, EZH2, RUNX1, ASXL1, PHF6, TP53, SF3B1, SRSF2, U2AF1, ZRSR2, NRAS, CBL, SETBP1, ETV6, and JAK2. For the gene length longer than 150 bp, such as FLT3-ITD, alternative RT-PCR method was used for analysis.

We further classified the gene mutations into functional groups similar to those previously described as follows: [27–29]. DNA methylation and hydroxymethylation-related DNMT3A, TET2 and IDH1/2; RNA spliceosome—SF3B1, SRSF2, ZRSR2 and U2AF1; chromatin remodelling ASXL1, EZH2, BCOR and KMT2A; transcriptional deregulation—CEBPA, RUNX1 and WT1; activated signalling—NRAS, KRAS, CBL, KIT, JAK2 and FLT3-ITD.

### Statistical analysis

Patients with complete remission (CR) after induction chemotherapy were defined according to the criteria of the International Working Group [30]. The discrete categorical variables of patients with and without specific molecular alteration were compared by using Fisher exact test, whereas continuous variables between groups were compared by using Mann–Whitney test. All statistical analyses were performed by using SPSS version 21 software (IBM Corp., Armonk, NY, USA) and considered *p*-values of less than 0.05 to be statistically significant.

## Results

### Patient cohort: clinical characteristics

A total of 93 de novo MDS patients were retrospectively summarized (Table 1), which included 62 patients with MDS-MLD, 1 patient with MDS-RS-SLD, 3 patients with MDS-RS-MLD, 8 patients with MDS-EB1, 19 patients with MDS-EB2. The median age at diagnosis was 55 years (range 15–85 years). Fifty patients were 59 years or younger with a median age of 44 (range 15–58) and 43 patients were 60 years or older with a median age of 68 (range 60–85) at diagnosis.

**Table 1:** Clinical manifestations and cytogenetic abnormalities of MDS patients stratified by age groups

	Total patients (n = 93)	Younger MDS patients (n = 50)	Older MDS patients (n = 43)	*p* value
Age	46 (16–87 years)	43 (16–59 years)	69 (60–87 years)	N/A
Gender males (n, %)	56 (60.2%)	32 (64.0%)	24 (55.8%)	0.421
WBC (×10^9^/L) (mean, range)	7.9 (0.3–123.9)	5.3 (0.7–123.9)	3.9 (0.3–11.1)	0.592
Hb (g/L) (mean, range)	79.1 (26.8–163.0)	80.1 (26.8–163.0)	77.9 (39.0–122.0)	0.701
Plate (×10^9^/L) (mean, range)	75.9 (4.0–233.0)	71.9 (4.0–233.0)	80.4 (3.0–586.0)	0.661
Blast in BM (mean, range)	5.5 (0.2–18.9)	4.1 (0.2–16.4)	7.1 (0.4–18.9)	0.007**

**Statistically singnificant difference (*p* < 0.01) was obseved between two groups

A total of 325 newly diagnosed AML patients, including 176 males and 149 females, were observed in this study. The clinical characteristics of these patients were summarized in Table 2. The median age at diagnosis was 46 years (range 16–87 years). 57 patients were 60 years or older with a median age of 69 years (range 60–87 years). 268 patients were 59 years and younger with a median age of 43 years (range 16–59). 19 patients were diagnosed as secondary AML (14 from myelodysplastic syndrome, 3 from CMML, 1 from chronic myeloid leukemia, and 1 with myelofibrosis). Among the 19 secondary AML patients, 6 were older patients and 13 were younger patients. There was no statistically significant difference in gender, white blood cells (WBC) or blasts in the bone marrow between younger patients and older patients.

**Table 2:** Clinical manifestations and cytogenetic abnormalities of AML patients stratified by age groups

	Total patients (n = 325)	Younger AML patients (n = 258)	Older AML patients (n = 57)	*p* value
Age	46 (16–87)	43 (16–59 years)	69 (60–87 years)	N/A
Gender males (n, %)	164 (53.8%)	134 (53.4%)	30 (55.6%)	0.772
WBC (×10^9^/L) (mean, range)	37.3 (0.1–450.7)	36.5 (0.1–450.7)	39.7 (0.5–316.1)	0.724
Hb (g/L) (mean, range)	78.6 (3.3–138.5)	78.0 (3.3–135)	81.0 (39–138.5)	0.384
Plate (×10^9^/L) (mean, range)	63.5 (2.0–442)	63.1 (2.0–442.0)	65.6 (3.0–376.0)	0.814
Blast in BM (mean, range)	57.4 (20.0–97.2)	58.1 (20.0–97.2)	54.3 (22–93.2)	0.275

### Molecular gene mutations of patients

Chromosome data of 183 AML patients was available at diagnosis, including 152 younger patients and 31 older patients (Table 2). Overall, there were 73 patients (39.9%) with a normal karyotype and 110 patients (60.1%) with a complex karyotype. 27 patients (14.8%), which included 25 younger patients and 2 older patients, carried t(8;21)(q22;q22.1) or RUNX1–RUNX1T1 gene fusion, while 5 patients (2.7%) carried inv(16)(p13.1q22) or t(16;16)(p13.1;q22) or CBFβ–MYH11 gene fusion, 6 patients (3.3%) carried + 8. Younger patients had a trend of higher incidence of t(8;21)(q22;q22.1) gene mutation (16.5% vs 6.5%, p = 0.153).

Among 93 MDS patients, at least one non-synonymous gene mutation was detected in 85 patients (91.4%) and no gene mutations were detected in 8 patients (8.6%). The median number of gene mutations was 2 (range 0–5). The distributions of molecular gene mutations are shown in Table 3. Among the 50 younger patients, at least one non-synonymous gene mutation was detected in 46 patients (92.0%) and no gene mutations were detected in 4 patients (8.0%). Among the 43 older patients, at least one non-synonymous gene mutation was detected in 39 patients (90.7%) and no gene mutations were detected in 4 patients (9.3%).

**Table 3:** Cytogenetic abnormalities of MDS patients stratified by age groups

	Total (n = 93)	Younger MDS patients (n = 50)	Older MDS patients (n = 43)	*p* value
Genes mutation total events (mean)	1.78	1.64	1.95	0.216
TET2	72 (77.4%)	41 (82.0%)	31 (72.1%)	0.255
ASXL1	21 (22.6%)	10 (20.0%)	11 (25.6%)	0.521
U2AF1	18 (19.4%)	10 (20.0%)	9 (20.9%)	0.912
RUNX1	9 (9.7%)	3 (6.0%)	6 (14.0%)	0.196
NRAS	7 (7.5%)	2 (4.0%)	5 (11.6%)	0.164
TP53	5 (5.4%)	2 (4.0%)	3 (7.0%)	0.526
SF3B1	5 (5.4%)	2 (4.0%)	3 (7.0%)	0.526
DNMT3A	5 (5.4%)	2 (4.0%)	3 (7.0%)	0.526
CEBPA	5 (5.4%)	3 (6.0%)	2 (4.7%)	0.774
SRSF2	5 (5.4%)	0 (0%)	5 (11.6%)	N/A
ETV6	4 (4.3%)	3 (6%)	1 (2%)	0.384
SETBP1	3 (3.2%)	2 (4%)	1 (2%)	0.649
JAK2	2 (2.3%)	0 (0%)	2 (5%)	N/A
PHF6	1 (1.1%)	0 (0%)	1 (2%)	N/A
NPM1	1 (1.1%)	0 (0%)	1 (2%)	N/A
IDH2	1 (1.1%)	0 (0%)	1 (2%)	N/A
EZH2	1 (1.1%)	1 (2%)	0 (0%)	N/A
CBL	1 (1.1%)	1 (2%)	0 (0%)	N/A
DNA methylation (frequency, %)	72 (77.4%)	41 (82%)	31 (72.1%)	0.547
RNA spliceosome (frequency, %)	28 (30.1%)	12 (24%)	16 (37.2%)	0.166
Chromatin remodelling (frequency, %)	22 (23.7%)	11 (22%)	11 (25.6%)	0.685
Transcriptional deregulation (frequency, %)	14 (15.1%)	6 (12%)	8 (18.6%)	0.375
Activated signalling (frequency, %)	10 (10.8%)	3 (6%)	7 (16.3%)	0.111

Among 325 AML patients, at least one non-synonymous gene mutation was detected in 279 patients (85.8%) and no gene mutations were detected in 46 patients (14.2%). The median number of gene mutations was 2 (range 0–7). The distributions of molecular gene mutations are shown in Table 4. Among the 268 younger patients, at least one non-synonymous gene mutation was detected in 229 patients (85.5%) and no gene mutations were detected in 39 patients (14.5%). Among the 57 older patients, at least one non-synonymous gene mutation was detected in 50 patients (87.7%) and no gene mutations were detected in 7 patients (12.3%).

**Table 4:** Cytogenetic abnormalities of AML patients stratified by age groups

	Total 325	Younger AML patients (n = 268)	Older AML patients (n = 57)	*p* value
Genes mutation total events (mean)	2.02	1.94	2.37	0.034*
TET2	164 (50.5%)	136 (50.7%)	28 (49.1%)	0.8238
ASXL1	62 (19.1%)	46 (17.2%)	16 (28.1%)	0.057^#^
CEBPA	56 (17.2%)	49 (18.3%)	7 (12.3%)	0.2758
FLT3	53 (16.3%)	45 (16.8%)	8 (14.0%)	0.609
DNMT3A	44 (13.5%)	35 (13.1%)	9 (15.8%)	0.584
NRAS	39 (12.0%)	32 (11.9%)	7 (12.3%)	0.943
NPM1	36 (11.1%)	29 (10.8%)	7 (12.3%)	0.7498
RUNX1	25 (7.7%)	17 (6.3%)	8 (14.0%)	0.0478*
IDH1	22 (6.8%)	19 (7.1%)	3 (5.3%)	0.618
IDH2	22 (6.8%)	13 (4.9%)	9 (15.8%)	0.0028**
KIT	19 (5.8%)	18 (6.7%)	1 (1.8%)	0.147^#^
ETV6	9 (2.8%)	7 (2.6%)	2 (3.5%)	0.7079
TP53	9 (2.8%)	4 (1.5%)	5 (8.8%)	0.0024**
WT1	8 (2.5%)	6 (2.2%)	2 (3.5%)	0.574
U2AF1	8 (2.5%)	6 (2.2%)	2 (3.5%)	0.574
PHF6	8 (2.5%)	6 (2.2%)	2 (3.5%)	0.574
EZH2	7 (2.2%)	7 (2.6%)	0 (0.0%)	NA
TTN	7 (2.2%)	5 (1.9%)	2 (3.5%)	0.4378
SF3B1	7 (2.2%)	3 (1.1%)	4 (7.0%)	0.005**
SRSF2	4 (1.2%)	3 (1.1%)	1 (1.8%)	0.693
JAK2	3 (0.9%)	2 (0.7%)	1 (1.8%)	0.4698
DNA methylation (frequency, %)	252 (77.5%)	202 (75.4%)	50 (87.7%)	0.0425*
RNA spliceosome (frequency, %)	20 (6.2%)	12 (4.5%)	8 (14.0%)	0.006399**
Chromatin remodelling (frequency, %)	69 (21.2%)	53 (19.8%)	16 (28.1%)	0.164379^#^
Transcriptional deregulation (frequency, %)	89 (27.04%)	72 (26.9%)	17 (28.8%)	0.649175
Activated signalling (frequency, %)	118 (36.3%)	101 (37.7%)	17 (28.8%)	0.262345

^#^Although there was difference, but no statistically significance was observed between two groups

*Statistically difference (*p* < 0.05) was obseved between two groups

**Statistically singnificant difference (*p* < 0.01) was obseved between two groups

The most common molecular event in the AML patient cohort was a TET2 (50.5%) mutation, followed by ASXL1 (19.1%), CEBPA (17.2%), FLT3-ITD (16.3%), DNMT3A (13.5%), NRAS (12.0%), NPM1 (11.1%), RUNX1 (7.7%), IDH1 (6.8%), and IDH2 (6.8%) mutations (Table 4). Meanwhile, the most common molecular event in the MDS patient cohort was a TET2 (77.4%) mutation, followed by ASXL1 (22.6%), U2AF1 (19.4%), NRAS (7.5%), TP53 (5.4%), SF3B1 (5.4%), DNMT3A (5.4%), CEBPA (5.4%) and SRSF2 (5.4%) mutations (Table 3).

AML patients had a significantly higher incidence of CEMPA (17.2% vs 5.4%, p = 0.0043), FLT3-ITD (16.3% vs 0.0%, p = 0.000), DNMT3A (13.5% vs 5.4%, p = 0.030962), NPM1 11.1% vs 1.1%, p = 0.002752), IDH1/IDH2 (1.5% vs 1.1%, p = 0.000628) gene mutation and significantly lower incidence of TET2 (48.3% vs 77.4% p = 0.000001) and U2AF1 (2.5% vs 19.4%, p = 0.000) gene mutations (Table 5) compared with MDS patients.

**Table 5:** Comparison of cytogenetic abnormalities between MDS and AML patients

	Total 325	Total 93	p value
Genes mutation total events (mean, %)	2.02	1.78	0.147^#^
TET2	164 (50.5%)	72 (77.4%)	0.000001**
ASXL1	62 (19.1%)	21 (22.6%)	0.4551
CEBPA	56 (17.2%)	5 (5.4%)	0.0043**
FLT3	53 (16.3%)	0 (0.0%)	0.000**
DNMT3A	44 (13.5%)	5 (5.4%)	0.030962*
NRAS	39 (12.0%)	7 (7.5%)	0.2242
NPM1	36 (11.1%)	1 (1.1%)	0.002752
RUNX1	25 (7.7%)	9 (9.7%)	0.536892
IDH1	22 (6.8%)	0 (0.0%)	0.000**
IDH2	22 (6.8%)	1 (1.1%)	0.000628**
KIT	19 (5.8%)	0 (0.0%)	0.000**
ETV6	9 (2.8%)	4 (4.3%)	0.0417*
TP53	9 (2.8%)	5 (5.4%)	0.217879
WT1	8 (2.5%)	0 (0.0%)	0.000**
U2AF1	8 (2.5%)	18 (19.4%)	0.000**
PHF6	8 (2.5%)	1 (1.1%)	0.417
EZH2	7 (2.2%)	1 (1.1%)	0.503
TTN	7 (2.2%)	0 (0.0%)	N/A
SF3B1	7 (2.2%)	5 (5.4%)	0.101
SRSF2	4 (1.2%)	5 (5.4%)	0.015
JAK2	3 (0.9%)	2 (2.2%)	0.337
DNA methylation (frequency, %)	252 (77.5%)	72 (77.4%)	0.980646
RNA spliceosome (frequency, %)	20 (6.15%)	28 (30.1%)	0.000**
Chromatin remodelling (frequency, %)	69 (21.2%)	22 (23.7%)	0.617282
Transcriptional deregulation (frequency, %)	89 (27.4%)	14 (15.1%)	0.014963*
Activated signalling (frequency, %)	118 (36.3%)	10 (10.8%)	0.000002**

^#^Although there was difference, but no statistically significance was observed between two groups

*Statistically difference (*p* < 0.05) was obseved between two groups

**Statistically singnificant difference (*p* < 0.01) was obseved between two groups

Between MDS and AML patients, AML patients had a significantly higher incidence of transcriptional deregulation (27.4% vs 15.1%, p = 0.014963) and activated signalling (36.3% vs 10.8%, p = 0.000002) related gene mutations and a significantly lower incidence of RNA spliceosome (6.2% vs 30.1%, p = 0.000) related gene mutations (Table 5).

Both the distribution of gene mutations and the pattern of mutation co-occurrence appear to be distinct between older and younger AML patients (Table 3). The mean number of molecular gene mutations at diagnosis was higher in older patients than younger patients (2.37 vs 1.94, p = 0.034). Older patients also had a significantly higher frequency of RUNX1 (13.0% vs 6.8%, p = 0.159), TP53 (9.3% vs 1.6%, p = 0.001) and IDH2 (16.7% vs 5.2%, p = 0.007) gene mutations and a trend of higher frequency of ASXL1 (28.1% vs 17.2%, p = 0.057) gene mutations.

Although there was a trend of higher incidence of gene mutation in the older group of MDS patients, no statistical significance was shown compared with the younger group (1.95 vs 1.64, p = 0.216). No statistically significant difference among the individual gene mutations among two groups of MDS patients was found.

Among the different gene functional groups in AML patients, the older patients had a significantly higher incidence of DNA methylation- and hydroxymethylation-related genes mutations (87.7% vs 75.4%, p = 0.0425) and RNA spliceosome (14.0% vs 4.5%, p = 0.0064). While there was a higher incidence of chromatin remodelling (28.1% vs 19.8%, p = 0.164) gene mutations, there was no difference in transcriptional deregulation (29.8% vs 26.9%, p = 0.649) and activated signalling (29.8% vs 37.7%, p = 0.262) related gene mutations. Meanwhile, no statistically significant difference was found between the older and younger groups of MDS patients. AML patients had a lower incidence of RNA spliceosome related gene mutations (6.15% vs 30.1%, p = 0.000) and a significantly higher incidence of transcriptional deregulation (27.4% vs 15.1%, p = 0.014963) and activated signalling (36.3% vs 10.8%, p = 0.000002) related gene mutations.

### Correlations between mutations and clinical outcomes of AML patients

Fifty-two patients returned to their local hospital for further chemotherapy after diagnosis in our center. In total, 273 patients received induction chemotherapy. Among these patients, 98 patients received standard DA regimen (Daunorubicin 60 mg/m^2^ per day on days 1–3 and Cytarabine 100 mg/m^2^ twice per day on days 1–7) and 47 patients received standard IA regimen (Idarubicin 12 mg/m^2^ per day on days 1–3 and Cytarabine 100 mg/m^2^ twice per day on days 1–7) as induction chemotherapy.

In the group of 98 patients received DA regimen, 56 (57.1%) patients who achieved CR after one course induction treatment. Meanwhile, in the group of 47 patients who received IA regimen, 36 (76.6%) patients achieved CR after one course induction treatment. When comparing these two different induction chemotherapy regimens, there was a significantly higher CR rate among the patients who received the IA regimen (76.6% vs 57.1%, p = 0.0228).

Among 11 patients with no gene mutation who received DA as the induction therapy, 8 patients (72.7%) achieved CR after one course induction treatment. Meanwhile, among 87 patients with one or more gene mutations who received DA induction therapy, only 48 patients (54.5%) achieved CR after one course induction treatment.

Among 13 patients with no gene mutation who received IA regimen as the induction therapy, 11 patients (84.6%) achieved CR after one course induction chemotherapy. Meanwhile, among 34 patients with one or more gene mutations who received IA induction therapy, only 25 patients (73.5%) achieved CR after one course induction treatment. (84.6% vs 73.5%, p = 0.442).

Nine older patients received either DA or IA regimen for induction chemotherapy. Five achieved CR (55.6%) after 1 course induction chemotherapy. Meanwhile, among the 136 younger patients received either DA or IA regimen chemotherapy, 87 patients achieved CR (64%) after 1 course induction chemotherapy. Younger patients showed no statistical significance in achieving CR compared to the older patients (64% vs 55.6%, p = 0.612).

## Discussion

NGS has opened new horizons for individualized diagnostics and therapy of myeloid malignancies including AML and MDS [1, 2]. In the past 5–10 years, NGS has been introduced in the most specialized hematologic laboratories with various myeloid NGS panels now being commercially available. Unlike the Sanger unit time detection single segment, NGS can simultaneously detect signals of thousands of channels, thus greatly improving efficiency. More and more genetic mutations in MDS and AML patients have been detected and these mutations may serve as potential markers to extend the prognostic parameters in AML. Detailed selection of targeted therapies can help us to explore more about the potential pathways or resistance mechanisms. Additional to the NGS, other methods had been used such as Restriction Fragment Length Polymorphism Analysis of PCR-Amplified Fragments (PCR–RFLP) and gel electrophoresis [31], the tetra-primer amplification refractory mutation system-polymerase chain reaction (ARMS-PCR) [32]. These are the simple and economical method to genotype single-nucleotide polymorphisms (SNPs) had been used as valuable tools for genotyping and genetic fingerprinting. The advantages and disadvantages of each tests are different due to the difference between the basis of each method, ARMS-PCR depend on primers and the set up performance, PCR–RFLP depend on the enzyme and the control sequence [31, 32]. Recently, a new high-resolution melting (HRM) analysis method, which was enzyme independence method and allowed variation screening in compare to other method for detecting mutation, had showed the cost-effectiveness and was able to show amount of the mutant allele carried in samples and it’s helpful for treatments follow-up and determining minimal residual disease in patients with myeloproliferative neoplasms. HRM analysis is an efficient and sensitive PCR-based approach for determining the gene mutation with capability to differentiate heterozygous and homozygous mutations [33]. Many believed that the sequencing is gold standard method in diagnosis of mutation. However, methods such as PCR–RFLP, ARMS-PCR and HRM can be very useful tools especially for the SNPs genotyping [31].

In our study, 91.4% of MDS patients and 85.8% of AML patients had at least one mutation detected by targeted NGS. This result is similar to some reports [15, 34]. Different gene mutation frequency results have been reported by different researchers [35–40]. These differences of the gene mutation frequencies could be due to the technical differences and algorithms for calling mutations. Therefore, we believe this detection can give useful genetic information that may be clinically applicable to current treatment methods.

Myelodysplastic syndrome (MDS) is clonal disorder characterized by ineffective hematopoiesis and a tendency to evolve into AML. Many genetic studies have identified a group of recurrently mutated genes contributing to the pathogenesis of MDS. These genes had been classified into a limited number of cellular processes, including RNA splicing, epigenetic and traditional transcriptional regulation, and signal transduction. The sequential accumulation of mutations drives disease evolution from asymptomatic clonal hematopoiesis to frank MDS, and, ultimately, to secondary AML [41]. Several large studies that have assessed the prognostic impact of MDS-associated gene mutations across a broad cross-section of patients [42–44]. Somatic mutations in certain genes reproducibly predict patient outcomes. Across studies, TP53, EZH2, ETV6, RUNX1, ASXL1, and SRSF2 mutations predict poor overall survival, whereas SF3B1 mutations are associated with better clinical outcomes. Interestingly, the prognostic significance of these mutations seems to be maintained regardless of whether these are early or late events in disease progression [43].

Considering the high incidence of mutations and cytogenetic alterations, it can be assumed that genomic instability plays a role in MDS pathogenesis [45]. Genomic instability is defined as the increased susceptibility of cells to acquire and spread genomic mutations or the inability of cells to deal with DNA damage [46]. There is growing evidence that the DNA damage response (DDR) or DNA repair machinery is impaired in MDS cells [47].

CEBPA was found to have the highest mutation rate for some cohorts in current literature [36, 37] as well as in some reports of Chinese AML patients [37, 38]. Our finding showed that TET2 had the highest gene mutation frequency (50.5%), followed by ASXL1 (19.1%), CEBPA (17.2%), FLT3–ITD (16.3%), DNMT3A (13.5%), NRAS (12.0%), NPM1 (11.1%), RUNX1 (7.7%) and IDH2 (6.8%) mutations. We detected NPM1 and FLT-ITD mutations at frequencies similar to the results reported by Hussaini et al. [39]. However, other groups have reported frequencies ranging from 20 to 33% [8, 19–23, 38]. Meanwhile, ASXL1 mutation frequencies were quite different from other reports, from 1% to 20% [8, 19–23, 39–41]. This difference may be due to the dissimilar patient populations. The most common molecular event in the MDS patient cohort was a TET2 (77.4%) mutation, followed by ASXL1 (22.6%), U2AF1 (19.4%), NRAS (7.5%), TP53 (5.4%), SF3B1 (5.4%), DNMT3A (5.4%), CEBPA (5.4%) and SRSF2 (5.4%) gene mutations. The results demonstrated the different gene mutation patterns between the AML patients and MDS patients. Other groups support our finding of similar gene mutation patterns and mutation frequencies between MDS and AML patients [48].

Although much effort has been made to clarify the correlation between molecular changes and clinical outcomes of AML patients, most of the gene mutation studies were among the younger patients. In our study, we analyzed the data from both young adults and older adults with AML. We confirmed that the frequency of molecular gene mutations at diagnosis was significantly higher in older patients than younger patients (2.37 vs 1.94, p = 0.034). Older patients had significantly higher frequency of RUNX1, TP53, IDH 2 and SF3B1 gene mutations. Additionally, older patients also had a trend of higher frequency of ASXL1 gene mutations. However, there was no significant difference between younger and older groups for MDS patients.

Gene mutations have been classified into different categories based on its functional groups previously by different studies: [27, 28, 30] DNA methylation and hydroxymethylation-related-DNMT3A, TET2 and IDH1/2; RNA spliceosome—SF3B1, SRSF2, ZRSR2 and U2AF1; chromatin remodeling—ASXL1, EZH2, BCOR and KMT2A; transcriptional deregulation—CEBPA, RUNX1 and WT1; activated signaling—NRAS, KRAS, CBL, KIT, JAK2 and FLT-ITD. Based on the classification, further analysis by gene mutation categories in AML patients showed that older AML patients had significantly higher incidence of DNA methylation and hydroxymethylation-related genes, RNA spliceosome (14.0% vs 4.5%, p = 0.0064) gene mutations, and a trend of higher incidence of chromatin remodelling (28.1% vs 19.8%, p = 0.164) gene mutations. However, there appears to be no difference between transcriptional deregulation and activated signalling related gene mutations in younger AML patients.

Comparing with the MDS patients, AML patients had a lower incidence of RNA spliceosome related gene mutations and a significantly higher incidence of transcriptional deregulation and activated signaling related gene mutations compared with MDS patients. However, no statistically significant difference of functional group related gene mutations between the age groups in MDS patients was found in our study. This clearly indicated that there were differences in the molecular status between MDS and AML patients. Biological studies and biochemical analyses of different variants have shed light on its dominant-negative and gain-of-function features in myeloid transformation via a variety of epigenetic changes. Based on these results, it would be possible to establish novel promising therapeutic strategies for myeloid malignancies harboring certain gene mutations such as ASXL1 by blocking interactions between ASXL1 and associating epigenetic regulators [28, 49].

Previously, TP53 and ASXL1 mutations were considered as poor prognostic factors [22, 29]. Our study confirmed TP53 and ASXL1 mutations are prevalent in both MDS and AML patients, especially in the older patients. The higher frequencies and burdens of unfavourable molecular mutations that are associated with poor prognosis in older patients might explain the dismal outcome in this patient group. Other reports had also demonstrated that as the number of oncogenic mutations increases, MDS patient outcomes progressively worsen [42, 43]. Recent studies had showed that cytogenetic and mutation tests for FLT3-ITD, NPM1 and CEBPA genes were meaningful for predicting outcomes in adult AML patients. Adverse cytogenetic abnormalities and FLT3-ITD mutation showed dismal RFS and OS [50].

The clinical practice of targeted NGS testing is useful for the identification of the AML patients who have an excellent chance of achieving a CR when treated with DA or IA induction chemotherapy. As for MDS patients, NGS technology can be used for diagnosis, classification, prognostication, disease surveillance and identification of patients suitable for targeted treatment. However, NGS data needs to further be interpreted and should be carefully used in the clinic and prospective clinical studies. This interpretation must be taken into consideration for aspects such as cytogenetic data and basic disease characteristics as well as other molecular issues (e.g. epigenetics and gene expression) [45].

## Conclusion

In conclusion, our data indicates gene mutations in MDS and AML patients that can be detected by NGS sequencing in majority of the patients (more than 85%). MDS and AML patients had different gene mutation patterns. There was a trend of gene mutation incidence increase from MDS-MLD to MDS-EB1/EB and AML. Older AML patients had higher frequencies and burdens of molecular mutations that are associated with poor prognosis and a lower incidence of favourable cytogenetics than younger patients. AML patients with fewer or no gene mutations had a better chance achieving CR with the IA and DA regimen for induction chemotherapy.

## Data Availability

Data and material will be available upon corresponding author approval. All data sets generated/analysed for this study are included in the manuscript and the additional files.
